# Chondroid and osseous metaplasia in an incidental type II papillary renal cell carcinoma with extensive solid areas: an unraveled molecular character

**DOI:** 10.11604/pamj.2018.31.26.16137

**Published:** 2018-09-13

**Authors:** Ayesha Ahmed

**Affiliations:** 1 Department of Pathology, College of Medicine, Imam Abdulrahman Bin Faisal University, Dammam, Kingdom of Saudi Arabia

**Keywords:** Renal cell carcinoma, papillary, cytogenetic

## Abstract

Chondroid and osseous metaplasia in a Type II Papillary renal cell carcinoma (PRCC) with extensive solid areas is a complex histological combination that has not been reported before. A 21 years old male presented with a comminuted fracture of right femur. On hematological investigations he was found to have low RBC count and hemoglobin. Radiological examination revealed an incidental, exophytic complex solid and cystic, mass lesion measuring 7 x 6.5 x 4.9 cm with thickened walls, septation and calcification. It was completely replacing upper pole of the left kidney with extension into perinephric fat. Enlarged para aortic and hilar nodes with necrosis were also noted. Radiological diagnosis was infectious processes such as tuberculosis versus malignancy. Surgical intervention comprising left partial nephrectomy was done. Histopathology and immunohistochemical analysis yielded the above diagnosis. Cytogenetic studies did not reveal gain of chromosome 7 and/or 17 or loss of chromosome Y, a characteristic genetic profile of PRCC. This case could be representative of a unique histological variant of PRCC in which the molecular profile yet needs to be unraveled.

## Introduction

Papillary renal cell carcinoma (PRCC) is the second most common type of renal cell carcinoma (RCC). Classically PRCC has been divided into two histological sub-types, 1 and 2. In the current era of cytogenetic and molecular classification of tumors, PRCC has shown much diversification. Characteristically the most common and characteristic chromosomal numerical aberrations seen in PRCC are the gains of chromosomes 7 and 17, and a loss of chromosome Y in male patients [1]. Many cases do not fit into these histological and cytogenetic framework and as such remain imprecisely classified. Here in we report such a case. The morphology was that of a type 2 PRCC with an addition of extensive solid foci which have rarely been previously reported. There also was an osseous and a chondroid metaplasia. Chondroid metaplasia in PPRC has never been reported before. The cytogenetic results did not show gain of chromosome 7 and/or 17 or loss of chromosome Y. Could this case represent a novel addition to a yet unraveled variant of PRCC?.

## Patient and observation

A 21 years old male presented with a post traumatic comminuted fracture in the proximal one third of right femur. In preparation for surgery his routine investigations were carried out. Chest x-ray revealed no cardiac, pulmonary or mediastinal abnormality. There was no joint space abnormality or foreign body detected in knee radiograph. The complete blood count and differential count were abnormal. The patient was anemic with RBC count 3.32mil/ul (4.7-6.1), hemoglobin 8.6 g/dl (13.0-18.0), hematocrit 26.0% (42.0-52.0) MCV 78.2 fL (80-94), MCH 25.9 pg (27-32) and MCHC 33.1 g/dl (32-36). Sickle cell screening was done and it was negative. His renal functions were unremarkable. Liver function tests were normal except for elevated SGOT 47u/l (15-37) and low levels of serum total proteins 5.2mg/dl (6.4-8.2) and serum albumin 2.4mg/dl (3.4-5.0). Random blood sugar was critically low, 47 mg/dl (≤140). CT scan abdomen and pelvis was advised. It showed an exophytic, complex solid and cystic mass lesion (7x6.5x4.9 cm, cranio-caudal, right-left and antero-posterior dimensions) with thickened walls and septae, replacing the upper pole of left kidney (Bosniak classification type IV). The solid components showed calcification. The lesion appeared to extend into the peri-nephric fat abutting the Gerota's fascia. There was no evidence of invasion into the adjacent organs. A rounded well defined left para aortic lymph node measuring 1.6 x 1.5cm with deeply hypodense center that could represent necrosis was present. A cystic lesion at the level of renal hilum with thick fluid density, possibly representing cystic lymph node was also seen. Another cystic lesion measuring 1.3cm, medial to the adrenal gland was also present. No other lesions were seen in the right kidney, bilateral pelvi-calyceal system, ureters, urinary bladder, pancreas, spleen or liver. Radiological diagnosis of the mass was malignancy versus an infectious process such as tuberculosis. Considering the patient's imaging findings and age, a pathological correlation was advised. The patient underwent left partial nephrectomy.

### Histopathology

**Gross examination:** Received in formalin was a partial nephrectomy specimen of the left kidney, weighing 1240 grams, with a grey brown smooth, glistening surface, measuring 14 x 6.5 x 3.7cm. The anterior surface was lobulated. Ureter measuring 6.5 x 0.4 cm was identified at the hilar area along with sutured blood vessels. On opening the kidney, a solid, cystic lesion measuring 7 x 6x 5cm was identified. The cysts were multiple and they ranged from 1-3cm in size. Two tan brown lymph nodes, one hilar and one suprahilar, measuring 2cm and 1.5cm in maximum dimensions, respectively, were also identified and sampled.

**Figure 1:**
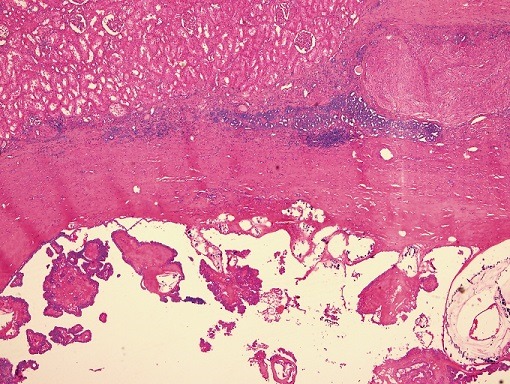
RCC showing cystic, papillary, solid areas along with normal renal tubules, (H&E 5x)

**Figure 2:**
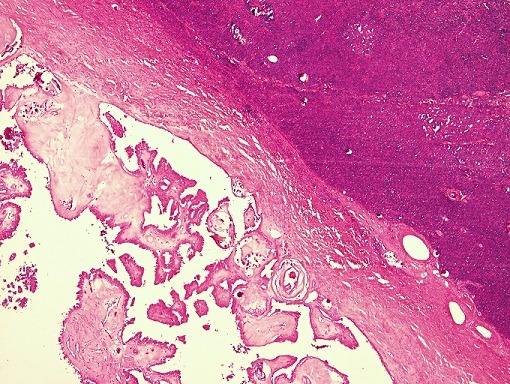
Interface between solid and papillary areas, (H&E 10x)

**Figure 3:**
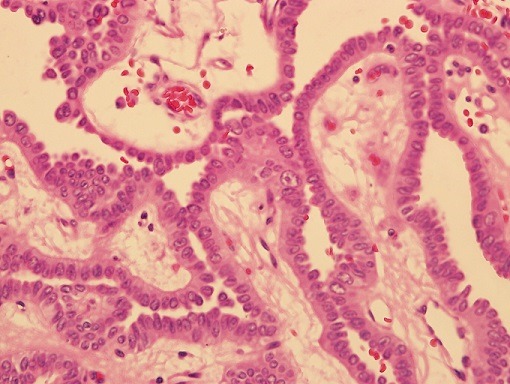
Papillary areas showing pleomorphic cells with eosinophilic cytoplasm and vesicular nuclei, (H&E 40x)

**Figure 4:**
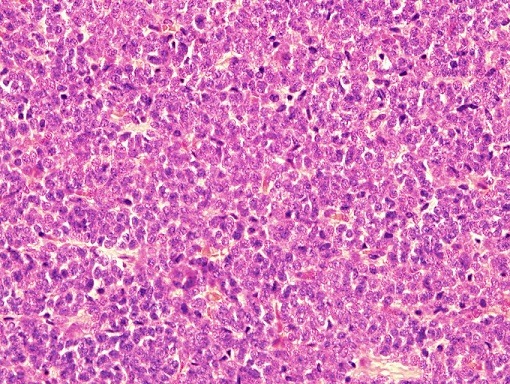
Solid areas showing sheets of monotonous small cells with hyperchromatic nuclei and scanty cytoplasm, (H&E 40x)

**Figure 5:**
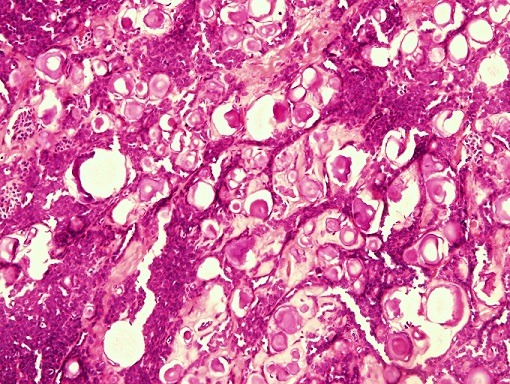
Extensive calcification, (H&E 40x)

**Figure 6:**
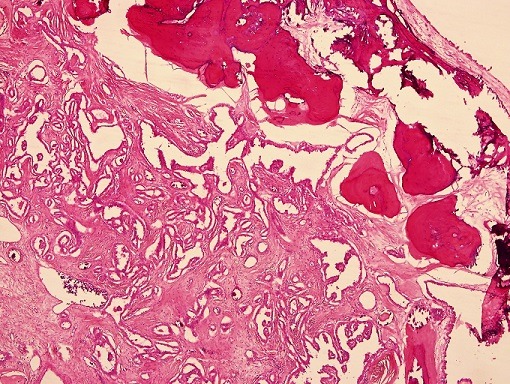
Osseous differentiation, (H&E 40x)

**Figure 7:**
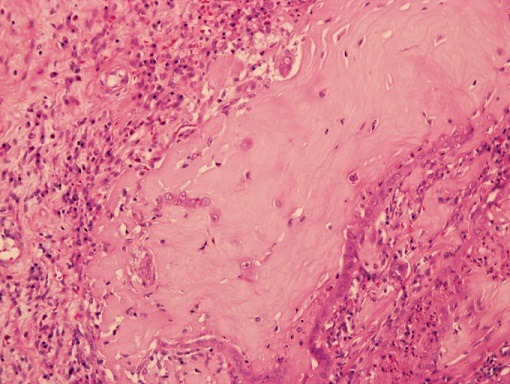
Chondroid differentiation, (H&E 40x)

**Microscopic examination:** Examined sections revealed a malignant tumor (Figure 1, Figure 2, Figure 3, Figure 4, Figure 5, Figure 6, Figure 7) comprising cysts, papillae and large foci of undifferentiated solid tumor showing extensive calcification, focal bone formation and cartilaginous differentiation. The cells lining the papillae and the cysts were eosinophilic, showing areas of stratification with vesicular, pleomorphic nuclei and prominent nucleoli. The solid areas showed diffuse sheets of blue cells with hyperchromatic, pleomorphic nuclei and scanty cytoplasm with foci showing abortive tubular formation. The tumor was seen extending into the peri-nephric fat. Lympho- vascular invasion was not identified. The two lymph-nodes sampled were seen to be completely replaced by the tumor.

**Immunocytochemistry:** The tumor cells were positive for Cytokeratin(CK) - 7, CK-18, CK-19, High Molecular Weight Cytokeratin (HMW-CK), AE1/AE3 and Vimentin, **A**lpha **M**ethyl **A**cyl **C**oenzyme A **R**acemase (AMACR), RCC marker, PAX-8 and Vimentin. These were negative for CD10, CD45, CD117, and Wilms Tumor (WT)-1. CK5/6, Melan A, HMB-45 and S-100.

**Cytogenetic studies:** There was no gain of chromosome 7 and/or 17 and also no loss of chromosome Y in both solid and papillary areas.

## Discussion

Many types of malignant tumors with varied morphological additives like ossification, calcification have been designated under the umbrella of Renal Cell Carcinoma (RCC). Herein, we report a unique combination of type II PRCC with peculiar features. It had extensive solid areas, osseous and, specifically, chondroid metaplasia and, cytogenetic studies that did not match with the classical alterations attributed to PRCC.

Regarding the solid foci, the term “solid type of PRCC” is designated when solid sheets of tumors cells with absence of true papillae are identified. The diagnosis is mainly dependent on immunohistochemical and cytogenetic analysis [2]. This variant is extremely rare. In these tumors an age of diagnosis ranging from 17 to 82 years with a male predominance, usually pT1, Fuhrman grade 2 and a favorable prognosis are documented [3]. The tumor in our case showed well defined, separate, solid and papillary areas. Ossification or metaplastic bone formation is infrequently seen in RCC. It has been reported in both benign and malignant renal tumors [4]. The renal lesions with osseous metaplasia include, ossifying renal tumor of infancy [5] lymphoma [6] and multiple variants of RCC such as clear cell [7, 8], chromophobe [9, 10], multilocular cystic [11], and papillary [8].

Multiple hypotheses have been postulated to explain bone formation in RCC. Bone morphogenetic protein, an inducer of osteoblastic differentiation of pluripotential stem cells, has been suggested to play a role in heterotopic bone formation in RCC. Under its influence, the neoplastic cells undergo osseous differentiation or metaplasia with production and deposition of dense collagenous matrix followed by mineralization and organization [12, 13]. Parathyroid hormone-related peptide has also been implicated in the progression of this process. Osseous metaplasia may represent a reparative or metaplastic response secondary to ischemia, necrosis, or inflammation in the tumor or surrounding tissues. It has been suggested that RCCs with calcification or bony metaplasia tend to be hypo-vascular and that this may predispose the tumor to ischemia and subsequent metaplasia [13]. Osseous metaplasia needs to be differentiated from osseous differentiation that occurs as a result of mesenchymal differentiation in tumors with a sarcomatoid component. This sarcomatoid transition can occur when RCC cells lose their epithelial features, acquire mesenchymal differentiation and develop a pleomorphic, spindle-cell like morphology. Such RCC are categorized as sarcomatoid RCC´s which are highly aggressive malignant lesions [14]. Ossified RCC´s are known to impart a better surgical and prognostic clinical outcome [8]. Besides metaplastic bone and calcification in different variants of renal cell carcinoma including type 1 PRCC, the presence of other elements has been rarely reported. Bone marrow elements have been reported by Cribbs *et al.* [4]. The current case is unique as, to our knowledge, there has been no reported case of type II PRCC with chondroid metaplasia.

Recent genetic and molecular studies have elaborated the traditional classification of PRCC that initially comprised type 1 and 2. The common chromosomal aberrations in PRCC include a combination of gains of chromosomes 7 and 17 with loss of chromosome Y in male patients. In the last decade, many sub-types and variants of PRCC have emerged that do not follow this genetic picture. Type 1 PRCC seems to be a genetically uniform entity. However, the molecular features in type 2 are more heterogenous. In our case, there was no gain of chromosome 7 and/or 17 and also no loss of chromosome Y in both solid and papillary areas [1]. This case could be representative of PRCC variant in which the molecular profile yet needs to be fathomed.

## Conclusion

Chondroid metaplasia in a Type II Papillary renal cell carcinoma (PRCC) with extensive solid areas and metaplastic bone is a complex histological combination that has not been reported before. Cytogenetic studies did not reveal gain of chromosome 7 and/or 17 and a loss of chromosome Y, a characteristic genetic profile of PRCC. This case could represent a unique histological variant of PRCC in which the molecular profile yet needs to be unraveled.

## Competing interests

The author declares no competing interests.
